# A new insight into a thermoplastic microfluidic device aimed at improvement of oxygenation process and avoidance of shear stress during cell culture

**DOI:** 10.1007/s10544-022-00615-1

**Published:** 2022-03-11

**Authors:** Zohreh Sheidaei, Pooria Akbarzadeh, Nam-Trung Nguyen, Navid Kashaninejad

**Affiliations:** 1 Faculty of Mechanical and Mechatronics Engineering, Shahrood University of Technology, Shahroud, Iran; 2 Laboratory of Life Sciences Electronics, École Polytechnique Fédérale de Lausanne, 1015 Lausanne, Switzerland; 3 Queensland Micro- and Nanotechnology Centre, Nathan Campus, Griffith University, 170 Kessels Road, Brisbane, QLD 4111 Australia

**Keywords:** Oxygen concentration, Thermoplastic, Microfluidic, Numerical simulation

## Abstract

Keeping the oxygen concentration at the desired physiological limits is a challenging task in cellular microfluidic devices. A good knowledge of affecting parameters would be helpful to control the oxygen delivery to cells. This study aims to provide a fundamental understanding of oxygenation process within a hydrogel-based microfluidic device considering simultaneous mass transfer, medium flow, and cellular consumption. For this purpose, the role of geometrical and hydrodynamic properties was numerically investigated. The results are in good agreement with both numerical and experimental data in the literature. The obtained results reveal that increasing the microchannel height delays the oxygen depletion in the absence of media flow. We also observed that increasing the medium flow rate increases the oxygen concentration in the device; however, it leads to high maximum shear stress. A novel pulsatile medium flow injection pattern is introduced to reduce detrimental effect of the applied shear stress on the cells.

## Introduction

In recent years, microfluidic technology has been meeting the need for a more realistic *in vitro* cell culture as a prerequisite for biological studies, drug testing, and tissue engineering (Fung et al. 2009; Kashaninejad et al. 2018; Nguyen et al. 2017). The development of various human-relevant cell culture platforms can address critical biological questions and decrease the need for animal research. Moreover, the deployment of microfluidic technology has led to a low consumption of resources, resulting in low research costs (Zhang and Ozdemir 2009). Despite all these advantages, preparing a microfluidic device for cell culture has its own challenges (Munaz et al. 2016). To keep cells healthy and actively growing, several critical factors should be simultaneously taken into account (Sheidaei et al. 2020). The availability of oxygen and other nutrients is one of these factors. Due to the small size of microchannels, oxygen supply to cell culture is limited, and oxygen can be quickly consumed. As such, it is highly desired to improve oxygen delivery in microfluidic cell culture platforms.

The implementation of numerical techniques in analyzing complex biological systems has recently increased significantly (Sheidaei et al. 2020). Utilizing numerical simulation as the first step of a research process provides a better understanding of the problem and guides the subsequent associated experimental analyses. Despite the importance of oxygen level in cellular microfluidic devices, the total number of related published articles over the past decade (2010–2020) is only approximately 600, which is not as expected (based on the analysis in the SCOPUS database; Search terms “microfluidic” AND “cell” AND “oxygen” in the title, abstract and keywords; Document type: articles only). The cost and difficulties of experimental analyses can be one of the reasons for this shortage. Although numerical analysis as a complementary method can help researchers with a deep understanding of oxygen concentration distribution within the device and effectively optimize the involved parameters to prevent the hypoxic condition, the number of relevant numerical studies is not adequate.

Mass transfer in microfluidic devices occurs as a result of diffusion and convection (Kim et al. 2013). Thus, depending on the type and density of cells, dimensional and material optimizations of the device and perfusing a sufficient medium flow can significantly affect the available oxygenation concentrations (Anada et al. 2012). To counteract oxygen depletion, it is common to use oxygen-permeable polymers for making microfluidic devices. Polydimethylsiloxane (PDMS) is a well-known material that is widely used in cellular studies due to its unique properties (Toepke and Beebe 2006). However, disadvantages of PDMS such as adsorption of small hydrophobic species led to the employment of other polymers with lower oxygen permeability, e.g., cyclic olefin copolymer (COC), poly(methacrylic acid), and poly(methyl pentene) (PMP) (Abaci et al. 2012; Mukhopadhyay 2007; Pu et al. 2007). Generally, it would be better to examine the role of material type on the oxygen level in a microfluidic device based on the definition of biological problems. Considering two different cell types, Ochs et al. (2014) used an adaptably designed microfluidic device to investigate the impact of three materials (PDMS, PMP, and COC) on the oxygen level in the absence of media flow rate. The team found that oxygen concentration decreases over time as oxygen diffusion is sufficiently slow through COC devices, especially for cells with a high oxygen consumption rate. Accordingly, the authors suggested applying a controlled media flow rate to counteract the oxygen reduction.

Injection of a sufficient media flow through a microfluidic device using a micropump is a possible solution for supplying the required oxygen to the cultured cells. Kheibary et al*.* (2020) utilized a 2D rectangular model to analytically study the effect of media flow rate on the oxygen supply level in microfluidic devices with oxygen-permeable and oxygen-impermeable membranes. The results indicated that applying a media flow with sufficiently high velocity reduces the effect of permeable membrane on channel oxygenation. Therefore, it would be possible to use an oxygen-impermeable membrane by applying a sufficient flow of media. However, the fluid-induced shear stress exerted on the cell layer can negatively affect cells’ function and behavior (Yu et al. 2014). As such, the media flow rate should keep the device’s oxygen level at the desired value and not exceed the limit to result in intolerable shear stress. Since it is hardly possible to measure the fluid-induced shear stress experimentally, analytical and numerical approaches are increasingly used for this purpose (Ip et al. 2016; Moghadas et al. 2018; Sheidaei and Akbarzadeh 2020). Moreover, numerical investigation of the role of media flow rates on the oxygen concentration within a microfluidic chip can be beneficial (Barisam et al. 2018; Du et al. 2017; Hu and Li 2007). Barisam et al. (2018) used a finite element method to determine the effect of various media flow rates on oxygen concentration distributions and fluid-induced shear stress around a trapped spheroid in a U-shaped microbarrier. The authors found that although increasing the media flow rate increases both the oxygen concentration and the applied maximum shear stress, the enhancement of oxygen level would be less effective for higher flow rates.

The dimensional properties of microfluidic devices are another parameter that can efficiently affect oxygen concentration distribution. Fabrication of microdevices with various sizes to find the optimized dimensions would be laborious and overwhelmingly expensive. The utilization of numerical methods for such an oxygen analysis could be beneficial; however, this approach has received less attention (Yu et al. 2017; Zahorodny-Burke et al. 2011). In a 2D model, the oxygen level as a function of the thickness of the PDMS membrane and the height of the media channel has been investigated in the literature (Zahorodny-Burke et al. 2011). The results showed that oxygen concentration at the bottom of the media channel decreases with increasing membrane thickness and channel height.

This study aims to employ 3D numerical simulations to comprehensively understand oxygen regulation in a complex cellular microfluidic device considering simultaneous mass transfer, medium flow, and cellular consumption. Despite the low oxygen diffusivity, COC is one of the frequently proposed materials in cell-based studies, mainly due to its potential for mass production (Pu et al. 2007). Therefore, the present study is devoted to deal with a popular commercial COC microfluidic device used by Ochs et al. (2014). Accordingly, the effect of media channel height and media flow rate is investigated on the oxygen concentration of the device during culture of two different cells. It should be emphasized that flow-induced shear rate and shearing time are two major parameters affecting the cell function. Therefore, they should be precisely considered in cell culture process. In the current study, in addition to the investigation of applied maximum shear stress (MSS) on the cell layer, a novel pulsatile flow injection model is represented innovatively to reduce the destructive impact of shear stress on the cells. In other words, the media flow will be injected through the device just when the oxygen depletion occurs, and thus the cells would experience a short-term shearing. Such a media injection pattern can provide the required oxygen to the cells without adversely affecting them.

The paper is structured as follows. Section 2 describes the geometrical model, assumptions, governing equations, boundary conditions, and simulation properties. This section also explains all possible states to model cell consumption to avoid confusion of fixed and variable consumption rates to help readers decide for their problem’s correct model. Section 3 compares the obtained results with the data represented in three relevant published studies to validate the numerical simulation. Section 4 examines the results under various conditions to optimize the device design in dimension and effectively deliver the flow rate.

## Material and methods

A commercial finite element software (COMSOL Multiphysics 5.5) was used to analyze the dissolved oxygen level in a 3D model during the culture of hepatocytes (HEP) and endothelial cells (EC). The varying oxygen concentration throughout the device is generally modeled using the module of transport of diluted species. This study is conducted in two steps: (i) considering no media flow to evaluate the effect of channel height on oxygen concentration and (ii) considering media flow to investigate the influence of flow rate and its injection pattern on oxygen concentration. As a critical parameter, medium-induced shear stress on the cells is also examined. Accordingly, a stop-and-flow media injection is proposed to reduce the destructive impact on the cells’ function.

### Geometry and cellular modeling

**Fig. 1:**
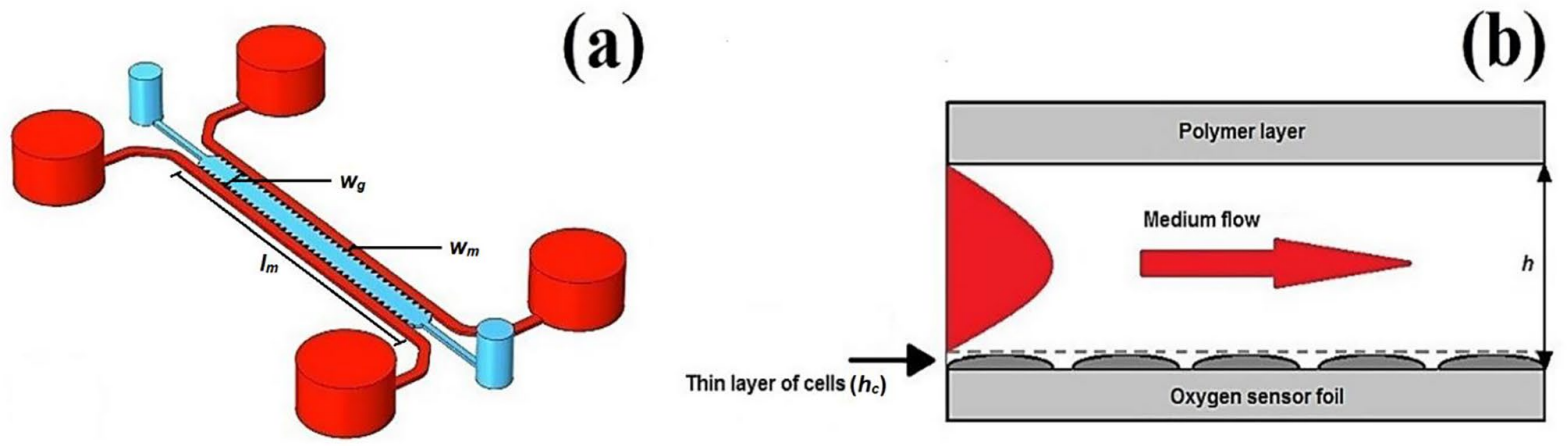
(**a**) Microfluidic chip design used in this study without considering the polymer layer surrounding the channels. The system consists of one central gel channel (blue) and two lateral media channels (red). (**b**) Cross-section of the media channel with the height of $h$. Cells are considered as a thin layer with the height of ${h}_{c}$ at the channel’s bottom. Here the polymer layer and oxygen sensor foil respectively on top of and under the media channel are shown to provide a general insight into the microchip structure

Figure 1a illustrates the geometry of the model considered in this study. The microfluidic chip consists of one central gel channel and two lateral media channels. The media channels on both sides of the gel channel, separated by micropillars and filled with media, supply the nutrients required for the growth and proliferation of the cultured cells. The hydrogel serves as a scaffold to for cells in a 3D tissue. According to the culture conditions reported by Ochs et al. (2014), the HEP and EC cells were individually seeded in the media channels and not in the gel channel. Our numerical model considers the cultured cells as a uniform thin layer on the bottom of the media channels (i.e., 2D culture). Figure 1b shows the schematic cross-section of the media channel. The chip is assumed to be made of COC polymer. As COC is oxygen-impermeable, the polymer layer around and on top of the gel and media channels are ignored to reduce the computational time. The polymer microfluidic device is placed on an oxygen sensor foil, which is also oxygen-impermeable. Chip geometry design and its dimensions are the same as the model represented in Ochs et al. (2014) study. Table 1 lists the key geometry parameters of the model.

**Table 1:** Geometry parameters of the microfluidic device

**Parameters**	**Values**
Height of the media channel ($h$)	150 $[\mathrm{\mu m}]$
Height of the cell sink layer ($${h}_{c})$$	10 $$[\mathrm{\mu m}]$$
Width of the media channels (${w}_{m}$)	500 $[\mathrm{\mu m}]$
Width of the gel channel (${w}_{g}$)	1.3 $[\mathrm{mm}]$
Length of the media channels (${l}_{m}$)	15 $[\mathrm{mm}]$

### Governing equations and boundary conditions

To assess the transient oxygen concentration throughout the microfluidic device, simulation is generally carried out by coupling the continuity (Eq. (1)), the Navier–Stokes (Eq. (2)), and the mass transfer (Eq. (3)) equations (Bird 2002). However, governing equations for different domains (i.e., gel, medium, cell, and pillars) are not the same, and one or more terms of (Eq. (3)) are eliminated according to the domain’s conditions. For example, the reaction term is considered just for the cell layer and the convection term is negligible in the absence of medium flow. Moreover, it should be noted that the hydrogel intrinsically is a porous material allowing passage of interstitial flow (Galie and Stegemann 2011). Nevertheless, since the velocity of the interstitial flow is very slow and results in a relatively small Peclet number (Pe ≪ 1), convection mass transfer in the hydrogels could be neglected. In the current study, the gel channel is located between two media channels with the same condition and pressure gradient. This configuration causes zero pressure gradient leading to very small interstitial fluid velocity compared to medium velocity in the lateral channels. Table 2 shows all possible governing equations for each domain.

1 $$\overrightarrow{\nabla }\cdot \overrightarrow{V}=0$$

2 $$\rho \left(\frac{d\overrightarrow{V}}{dt}+\overrightarrow{V}\cdot\overrightarrow{\nabla }\overrightarrow{V}\right)=-\overrightarrow{\nabla }p+\mu {\nabla }^{2}\overrightarrow{V}$$

3 $$\frac{\partial C}{\partial t}+\overrightarrow{V}\cdot \overrightarrow{\nabla }C=\overrightarrow{\nabla }\cdot \left(D\overrightarrow{\nabla }C\right)-R$$

where $\rho$ is the fluid density, $\overrightarrow{V}(x,y,z,t)$ is the velocity field, $p(x,y,z,t)$ is the pressure, $\mu$ is the fluid viscosity, $t$ is time, $C(x,y,z,t)$ is concentration, $D$ is the diffusion coefficient of oxygen, and $R(x,y,z,t)$ is the volumetric reaction term. It is noteworthy that the medium’s hydrodynamic properties are similar to those of water at $37\;^\circ{\rm C}$ (Naciri et al. 2008). Table 3 summarizes the values of simulation parameters. $R$ represents the oxygen consumption rate of cells and can be expressed either as a constant number or as a variable function of concentration. Michaelis–Menten equation (Eq. (4)) is one of the best-known approaches to model a nonlinear variable reaction rate (Buchwald 2009).

4 $$R(x,y,z,t)=\frac{C(x,y,z,t)}{C(x,y,z,t)+{K}_{m}}{\rho }_{cell}{V}_{\mathrm{max}}$$

where ${V}_{\mathrm{max}}$ is the maximum reaction rate, ${\rho }_{cell}$ is volumetric cell density, and ${K}_{m}$ is Michaelis constant. Moreover, based on experimental observations, other variable functions can be postulated to model the different oxygen consumption rate of cells (Chandel et al. 1997; Subramanian et al. 2007). For HEP cells, a linear concentration-based equation was suggested (Ochs et al. 2014):

**Table 2:** The governing equations for each domain of the model

**Domain**	**Governing equations**
Medium	Convection—diffusion equation
Cell layer	Convection—reaction—diffusion equation
Gel	Diffusion equation
Pillars	Diffusion equation

**Table 3:** Details of the parameters used for the numerical simulations. The values are based on the experimental results of Ref. (Ochs et al. 2014)

**Parameters**	**Values**	**Descriptions**
$\rho$	$1000\mathrm{kg}/{\mathrm{m}}^{3}$	Density of medium
$\mu$	$0.718\times {10}^{-3}\mathrm{Pa}.\mathrm{s}$	Viscosity of medium
${D}_{m}$	$3.35\times {10}^{-9}{\mathrm{m}}^{2}/\mathrm{s}$	Diffusion coefficient of ${O}_{2}$ through the medium
${D}_{c}$	$3.35\times {10}^{-9}{\mathrm{m}}^{2}/\mathrm{s}$	Diffusion coefficient of ${O}_{2}$ through the cell layer
${D}_{g}$	$3.35\times {10}^{-9}{\mathrm{m}}^{2}/\mathrm{s}$	Diffusion coefficient of ${O}_{2}$ through the gel
${D}_{p}$	$0{\mathrm{m}}^{2}/\mathrm{s}$	Diffusion coefficient of ${O}_{2}$ through pillars
${C}_{0.m}$	$17\%$	Initial oxygen concentration of the medium
${C}_{0.c}$	$17\%$	Initial oxygen concentration of the cell layer
${C}_{0.g}$	$17\%$	Initial oxygen concentration of the gel
${C}_{0.p}$	$0\%$	Initial oxygen concentration of the pillars
${V}_{max. EC}$	$2\times {10}^{-17}\mathrm{mol}/\mathrm{s}$	Maximum reaction rate of the EC cells
${\#}_{ EC}$	$11796$	Number of ECs

5 $$R(x,y,z,t)=0.0256C(x,y,z,t)-{10}^{-4}$$

Furthermore, in some cases, to a reduced-form of Eq. (4) is employed as a constant uptake rate simplify the study (Allen and Bhatia 2003; Tilles et al. 2001; Zahorodny-Burke et al. 2011):

6 $$R={V}_{max}{\rho }_{cell}$$

In other words, when the value of ${K}_{m}$ in comparison with the concentration is negligible, it is assumed that $C/\left(C+{K}_{m}\right)=1$.

In addition to 2D cell culture in the media channels, the device provides cell seeding within the gel channel to develop a realistic 3D cellular culture for cancer research, vascular biology, immuno-oncology, and neurobiology (Li et al. 2017; Shin et al. 2012; Xiao et al. 2019). A 3D cell culture in the gel allows tissue of various geometries (e.g., round, stellate, grape-like, and branching) to grow and interact with their surroundings similar to *in-vivo* conditions.

Although our current study only considers a 2D cell culture (modeled as a cuboid configuration) on the bottom of the media channel, the developed 3D numerical model considers concentration gradient in all three spatial dimensions can be applied to model cell culture in the gel channel. In this case, the reaction term $R(x,y, z, t)$ obtained from experiments is added to the governing equation of the gel domain. Note that the variable reaction rate is utilized identically for 2D and 3D cultures. However, based on the cell type and its configuration, the affecting parameters (e.g., cell density, maximum reaction rate, and Michaelis constant) are characterized to model the consumption rate of the desired cell in the microfluidic device. Furthermore, based on the location of the cultured cell and its surrounding environment and co-cultured cells, the local concentration $C(x,y, z, t)$ varies with its location and time ($x$, *y*, $z$, $t$).

In this study, a variable model (Eq. (5)) and a fixed model (Eq. (6)) were considered for HEP and EC cells’ oxygen consumption rate, respectively. One solution is to model only a portion of the device imposing the symmetry condition to reduce the calculation time. Since the geometry and boundary conditions are symmetric, only a quarter of the device was considered in the case without the media flow. Accordingly, in the case of media flow, a half model was implemented due to geometric symmetry. As COC and sensor foil are oxygen-impermeable, the no-flux boundary condition was used on all outer surfaces of the channels. A fixed oxygen concentration of $17\%$ was set to all boundaries exposed to the experimental environment. A defined flow rate at the inlet of the medium stream and a zero-pressure condition at the outlet were used to solve Eq. (2). The initial oxygen concentration of all domains is presented in Table 3. All domains were discretized using the tetrahedral mesh. The total number of elements for the quarter and half models are considered $\sim 2.2\times {10}^{5}$ and $\sim 3.9\times {10}^{5}$, respectively. Relative tolerance is set to $0.001$.

## Validation of the study

To validate the numerical diffusion–reaction solution, the transient oxygen concentration in the COC microfluidic device of both HEP and EC culture was compared with numerical and experimental data represented by Ochs et al. (2014), Fig. 2. Accordingly, boundary conditions, initial values, and geometric and material parameters were defined as the same. A constant concentration was assumed for the outer surfaces subjected to the external environment. A no-flux boundary condition was considered on the lower surface to represent oxygen-impermeable sensor foil. More details on the defined parameters could be found in Ref. (Ochs et al. 2014). In this simulation, the diffusion coefficient in COC was defined as zero. The results showed an excellent agreement between the present study and the solution of Ochs et al. (2014).

**Fig. 2:**
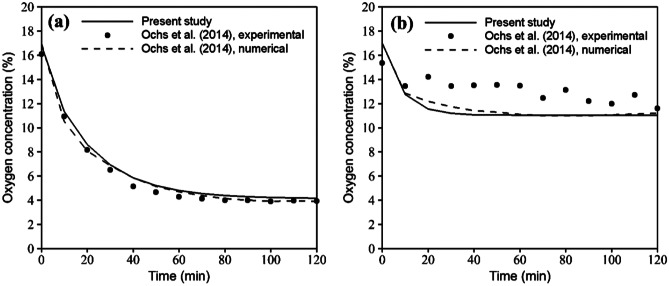
Comparison of the present study and numerical/experimental results of Ochs et al. (2014) for the transient oxygen concentration in the COC microfluidic device during (**a**) HEP and (**b**) EC cultures in the absence of medium flow

**Fig. 3:**
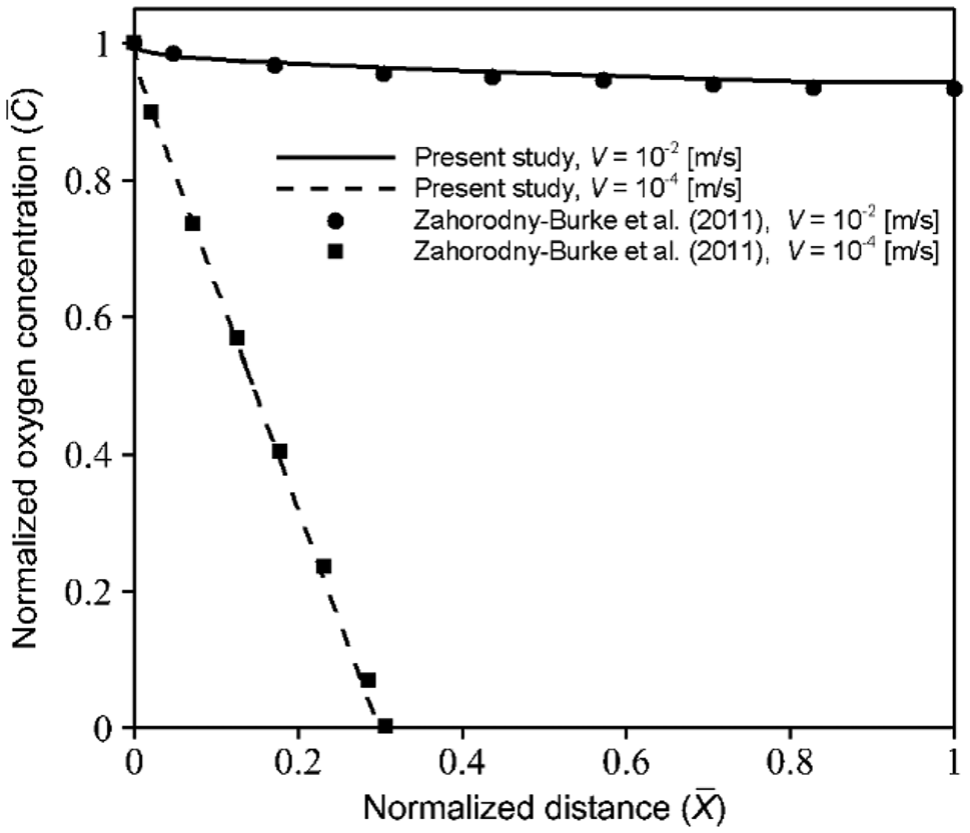
Comparison of the present study and numerical solution of Zahorodny-Burke et al. (2011) for the dimensionless oxygen concentration in a 2D rectangular microfluidic channel with two different average velocities

Moreover, to validate the convection–diffusion-reaction solution, the steady-state oxygen concentration in a 2D rectangular microchannel with the medium flow was examined, and the obtained results were compared to the data presented by Zahorodny-Burke et al. (2011). Components of this model were similar to those of the media channel considered in the present study (i.e., COC layer, media channel, and cell sink) (see Fig. 1b). The diffusion coefficient of the COC layer was considered to be close to zero. A constant model was defined for the cell oxygen consumption rate. For more details on the boundary conditions, initial values, cell consumption rate, and geometric and material properties, readers can refer to Ref. (Zahorodny-Burke et al. 2011). The distribution of dimensionless oxygen concentration ($\overline{C }$) as the function of normalized distance along the channel length ($\overline{X }$) for two different media average velocities ($V$( is illustrated in Fig. 3. Here $\overline{C }$ is the normalized oxygen concentration with respect to the saturation concentration of oxygen in the cell culture medium (${C}_{sat.media}=0.2\mathrm{mol}/{\mathrm{m}}^{3}$) and $\overline{X }$ is the normalized distance with respect to the channel length ($L=15\mathrm{mm})$ (Zahorodny-Burke et al. 2011). The comparison indicates an excellent agreement between the present study and the numerical solution of Ref. (Zahorodny-Burke et al. 2011).

**Fig. 4:**
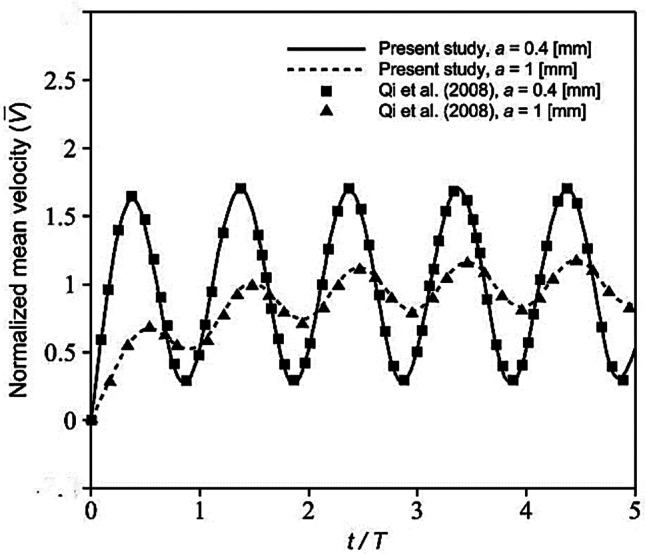
Comparison of the present study and numerical solution of Qi et al. (2008) for the transient response of fluid mean velocity to a sinusoidal pressure gradient

Since the main objective of this paper is studying the role of pulsed flow on the oxygen concentration in a cellular microfluidic device, a validation was presented for this kind of flow in a simple microchannel using an analytical study by Qi et al. (2008). For this purpose, the incompressible and Newtonian fluid flow in the channel under sinusoidal pressure gradient was investigated. The cross-section of the channel was considered square with two different dimensions of $a=0.4 \mathrm{mm}$ and $1 \mathrm{mm}$. Fluid density and viscosity were respectively defined equal to $1000 \mathrm{Kg}/{\mathrm{m}}^{3}$ and $1 \mathrm{mPa}.\mathrm{s}$. The obtained numerical results for the transient normalized mean velocity ($\overline{V }$) have a good agreement with the results of Qi et al. (2008) (see Fig. 4). To normalize the mean velocity, the results were divided by the steady state mean velocity. Accordingly, time was normalized using period of flow oscillation ($T=0.05 \mathrm{s}$).

## Results and discussion

A numerical model is used to predict and understand how the various conditions affect the oxygen concentration in the microfluidic device, towards effective optimization of geometries and applied flow rates. Thus, oxygen concentration in both HEP and EC culture for different channel heights and injection flow rates was examined. Moreover, the shear stress as a destructive effect of medium fluid flow on the cultured cells was investigated, and a pulsatile flow rate was introduced to reduce the shear stress. All results are presented in dimensionless form as follows:

7 $${t}^{*}=\frac{t{V}_{max.cell}}{{c}_{0.c}{v}_{cell}}$$

8 $${C}^{*}=\frac{C}{{C}_{0,m}}$$

9 $${Q}^{*}=\frac{Q}{{Q}_{min,ref}}$$

10 $${\tau }_{max}^{*}=\frac{{\tau }_{max}}{{\tau }_{cell,ref}}$$

11 $${\tau }_{ave}^{*}=\frac{{\tau }_{ave}}{{\tau }_{cell,ref}}$$

where ${t}^{*}$ is normalized time, ${C}^{*}$ is normalized concentration, ${Q}^{*}$ is normalized flow rate, ${\tau }_{max}^{*}$ is normalized MSS, and ${\tau }_{ave}^{*}$ is normalized average shear stress. Also ${v}_{cell}$ and ${\tau }_{max}$ are the cell volume and MSS, respectively. ${Q}_{min,ref}$ and ${\tau }_{cell,ref}$ are presented minimum flow rate and exerted shear stress on the cell layers as introduced by Ochs et al. (2014), respectively. For HEP and EC, ${Q}_{min,ref}$ is equal to $2.73 \times {10}^{-11}[{\mathrm{m}}^{3}/\mathrm{s}]$ and $3.3 \times {10}^{-12} [{\mathrm{m}}^{3}/\mathrm{s}]$, and ${\tau }_{cell,ref}$ is $1.57 \mathrm{Pa}$ and $0.19 \mathrm{Pa}$, respectively.

### Effect of media channel height on the oxygen concentration

**Fig. 5:**
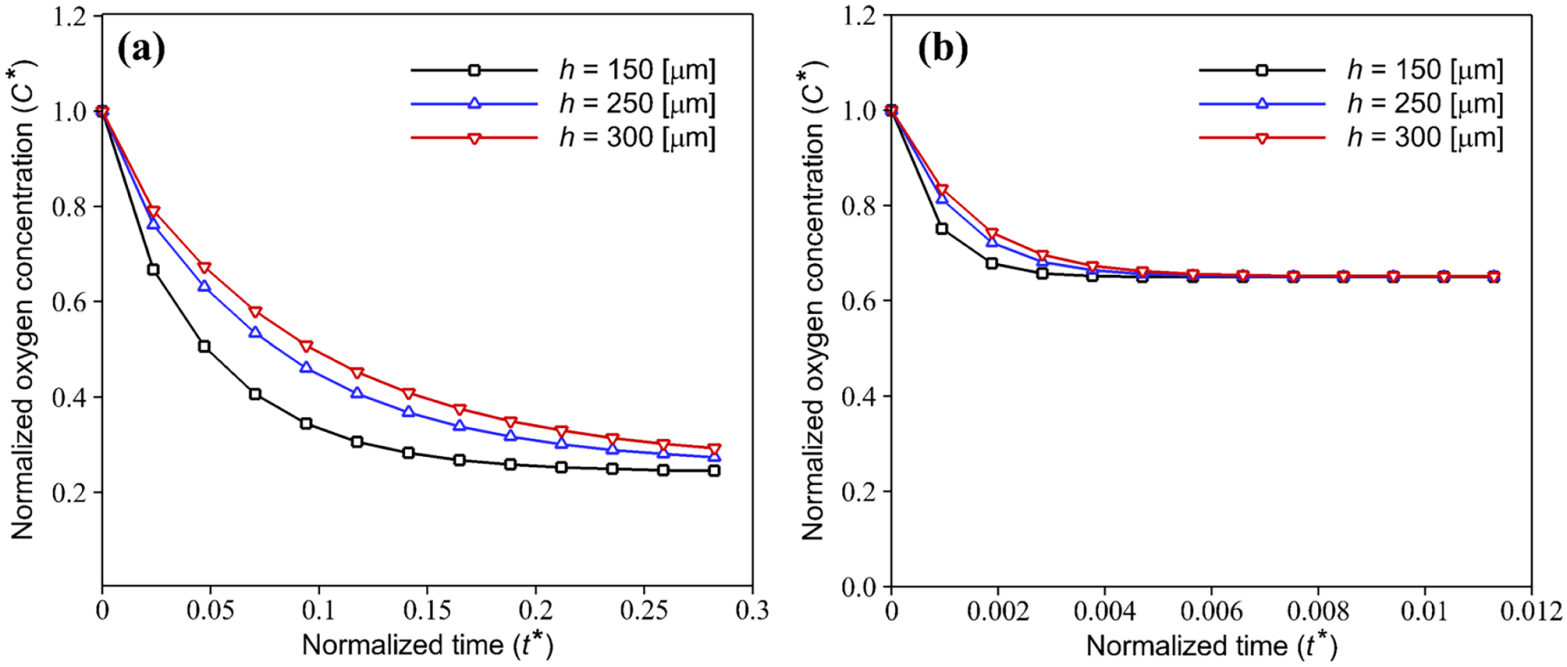
Effect of different media channel heights on normalized transient oxygen concentration in the COC microfluidic device during (**a**) HEP and (**b**) EC culture with varying and constant oxygen consumption rates, respectively

To determine the effect of media channel height on oxygen level, the normalized oxygen concentration in the microfluidic device with HEP and EC culture was plotted against the non-dimensional time for three different channel heights, Fig. 5. We assumed that the oxygen transfer in the device occurs just by diffusion. Therefore, as the COC is oxygen-impermeable, the media play a crucial role in supplying the required oxygen of cells. According to the consumption rate of cells, oxygen concentration decreases over time and thereafter reaches a constant value. Results for HEP indicate that increasing the channel height rises the required time for oxygen depletion. For instance, in a channel with a height of $300 \mathrm{\mu m}$, oxygen level down to $11\%$ occurs approximately $10 \mathrm{min}$ later than the channel with a height of $150 \mathrm{\mu m}$. Moreover, this delay is more sensible in low oxygen concentrations. However, for the case with EC culture, rising the channel height had no significant effect on oxygen concentration, and a slight increase was observed in the early times. So, it is predicted that increasing the media channel height could be an effective way to improve the oxygenation process, especially during the culture of cells with high oxygen consumption rates.

### Effect of medium flow rate on the oxygen level

**Fig. 6:**
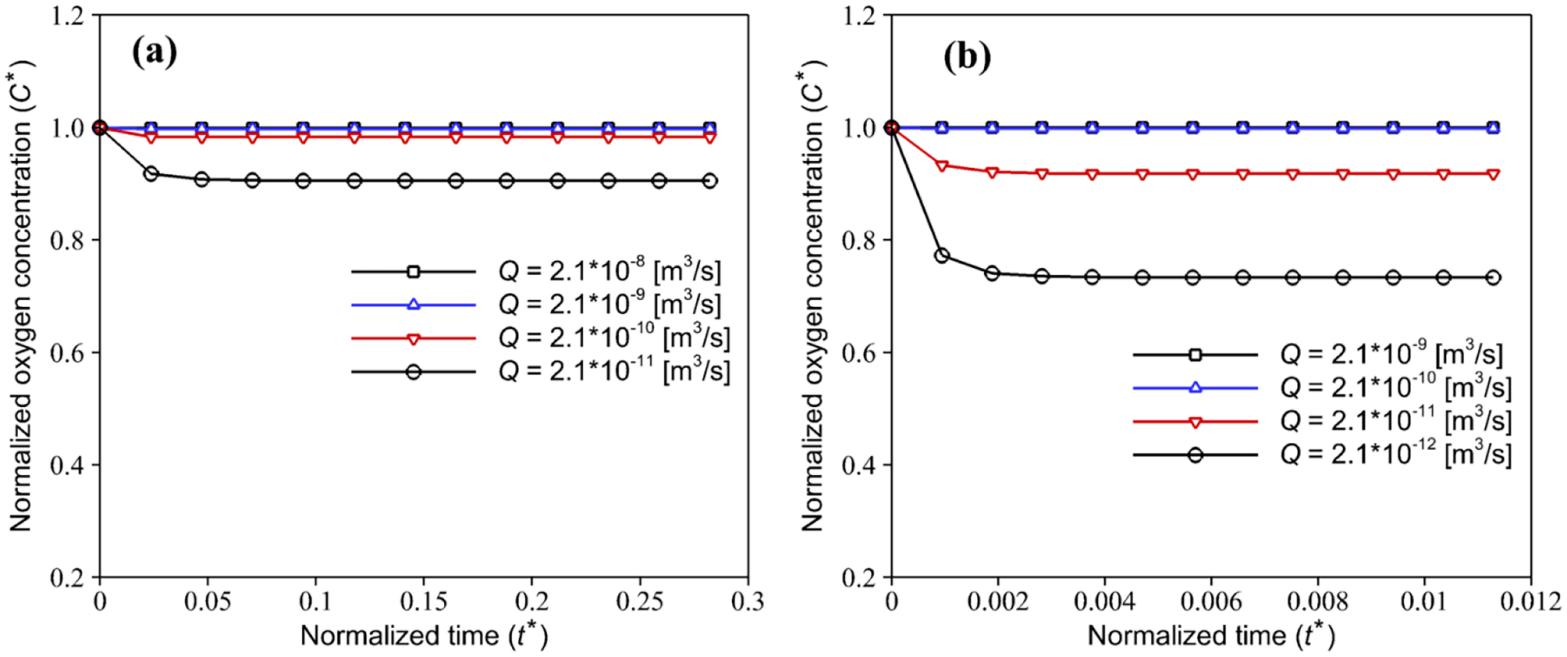
Effect of different medium flow rates on normalized transient oxygen concentration in the COC microfluidic device with a channel height of $150 \mu m$ during (**a**) HEP and (**b**) EC culture, respectively with variable and constant oxygen consumption rates

To prevent the hypoxic condition, a continuous medium flow was applied to the COC microdevice. Hence, the mass transfer would take place both by diffusion and convection. To find the sufficient medium flow rate, the oxygenation profiles of HEP and EC with various medium flow rates ($Q[{\mathrm{m}}^{3}/\mathrm{s}]$) are investigated (see Fig. 6). Results expectedly indicate that the volumetric flow rate can affect the oxygen concentration within the device. According to the numerical solution, a minimum flow rate of $2.1 \times {10}^{-9}[{\mathrm{m}}^{3}/\mathrm{s}]$ and $2.5 \times {10}^{-10} [{\mathrm{m}}^{3}/\mathrm{s}]$ should be injected into the media channel in order to compensate the oxygen depletion during the culture of HEP and EC, respectively. In this case, the flow rate in the HEP and EC layers is respectively equivalent to $2.73 \times {10}^{-11}[{\mathrm{m}}^{3}/\mathrm{s}]$ and $3.3 \times {10}^{-12} [{\mathrm{m}}^{3}/\mathrm{s}]$, which is in good agreement with the mathematical calculation of Ochs et al. (2014). For media flow rates greater than these values, the oxygen concentration in the device for both EC and HEP cell culture remains constant at the maximum value. Moreover, the effect of different medium flow rates on the oxygen concentration in a channel with the height of $h=300 \mathrm{\mu m}$ was analyzed (data not shown). It is observed that increasing the channel height has no significant effect on the oxygen concentration for a given medium flow rate, and oxygen transfer to the cells occurs primarily by convection rather than diffusion. However, in the case of a very low flow rate, a slight increase in the oxygen concentration was observed.

### Effect of medium flow rate and media channel height on the applied shear stress

**Fig. 7:**
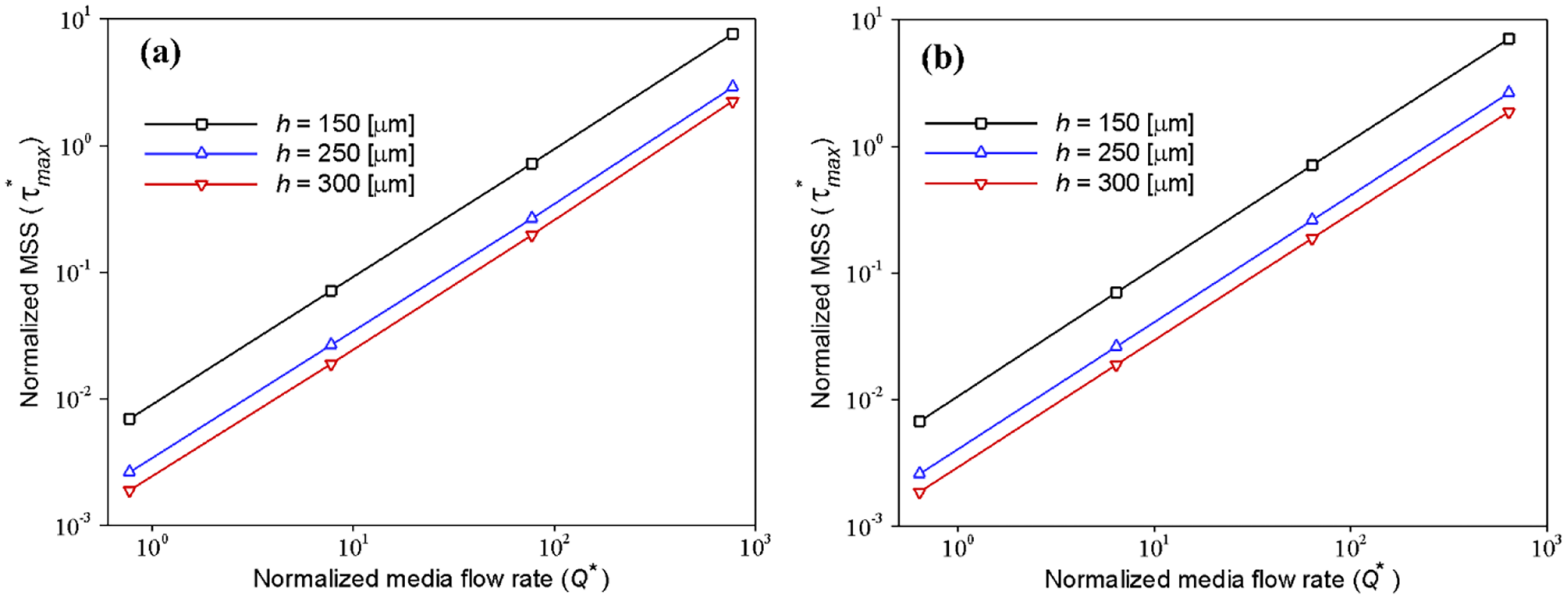
Effect of media flow rate and media channel height on the applied MSS on the (**a**) HEP and (**b**) EC cells’ layer in the COC microfluidic device, respectively, with variable and constant oxygen consumption rates. The diagrams are drawn on the logarithmic scale

During continuous perfusion, medium flow through the channel exerts shear stresses on the cell layer, which interfere with the behavior and function of cells. However, regarding the cell type, various impacts can be observed depending on the value of applied shear stress (Olivier and Truskey 1993). Therefore, researchers should find an optimized medium flow rate that not only provides adequate oxygenation but also protects cells from the destructive effects of shear stress. Geometry properties are one of the critical factors influencing the shear stress in the microchannels. Thus, this section investigates the impact of medium flow rates on the applied MSS in channels with three different heights, Fig. 7. Although all the obtained values of MSS for both HEP and EC cells are in an acceptable range (Ochs et al. 2014), reducing the shear stress will restrict its destructive effects. The previous section demonstrated that increasing the channel height does not affect the oxygen concentration during both EC and HEP culture. However, we observed that increasing the channel height could partly reduce the effective MSS on the cells for a given flow rate. Although increasing the media flow rate guarantees cultured cells’ oxygenation, it will cause higher MSS, which is harmful to the cells. Therefore, increasing the channel height can reduce the destructive effect of high medium flow rate.

### Effect of pulsatile medium flow injection on the oxygen concentration and shear stress

**Fig. 8:**
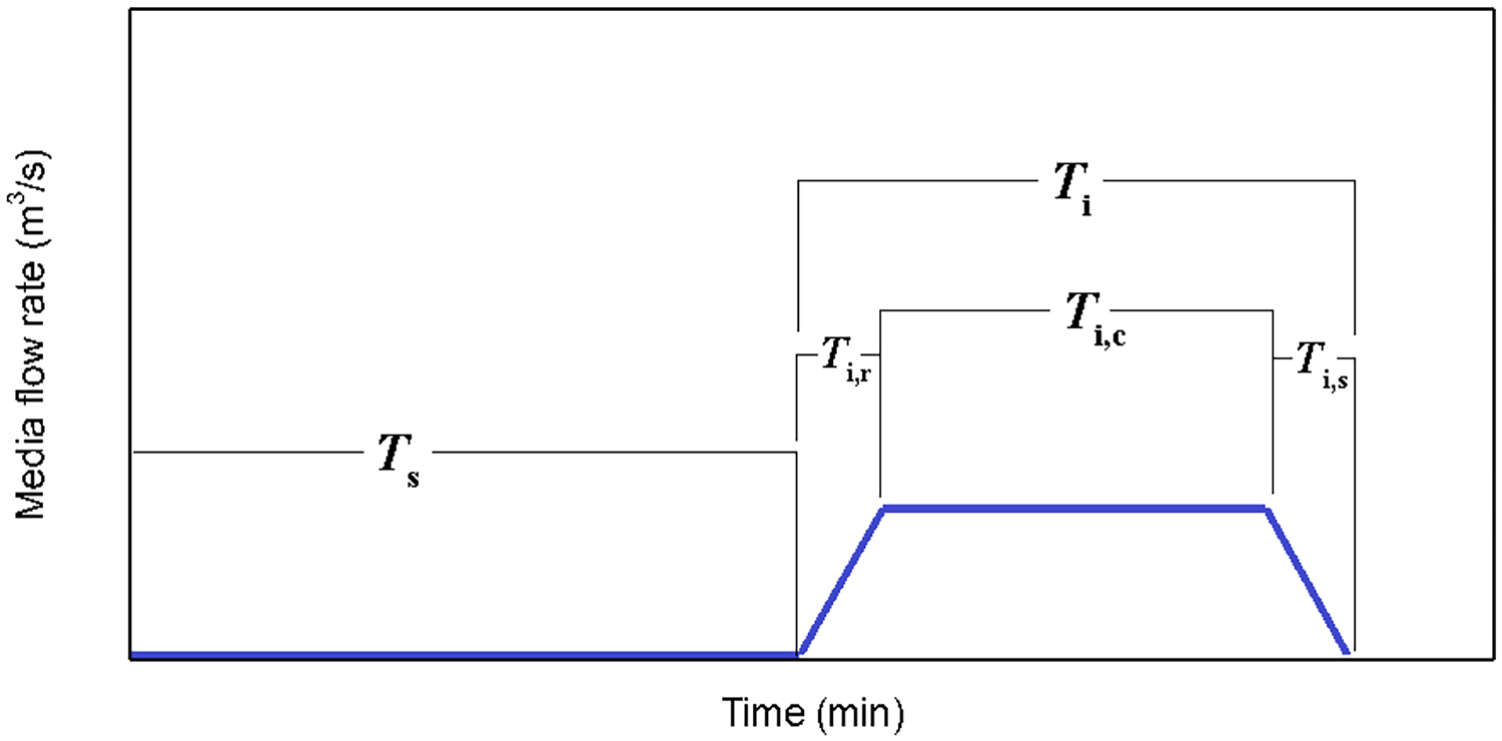
The cyclic pulsatile pattern for the medium flow injection through the COC microfluidic device. ${T}_{\mathrm{s}}$ is stagnation time, ${T}_{\mathrm{i}}$ is total injection time, ${T}_{\mathrm{i},\mathrm{c}}$ is continuity time, and ${T}_{\mathrm{i},\mathrm{r}}$ and ${T}_{\mathrm{i},\mathrm{s}}$ are rising and settling times, respectively

**Fig. 9:**
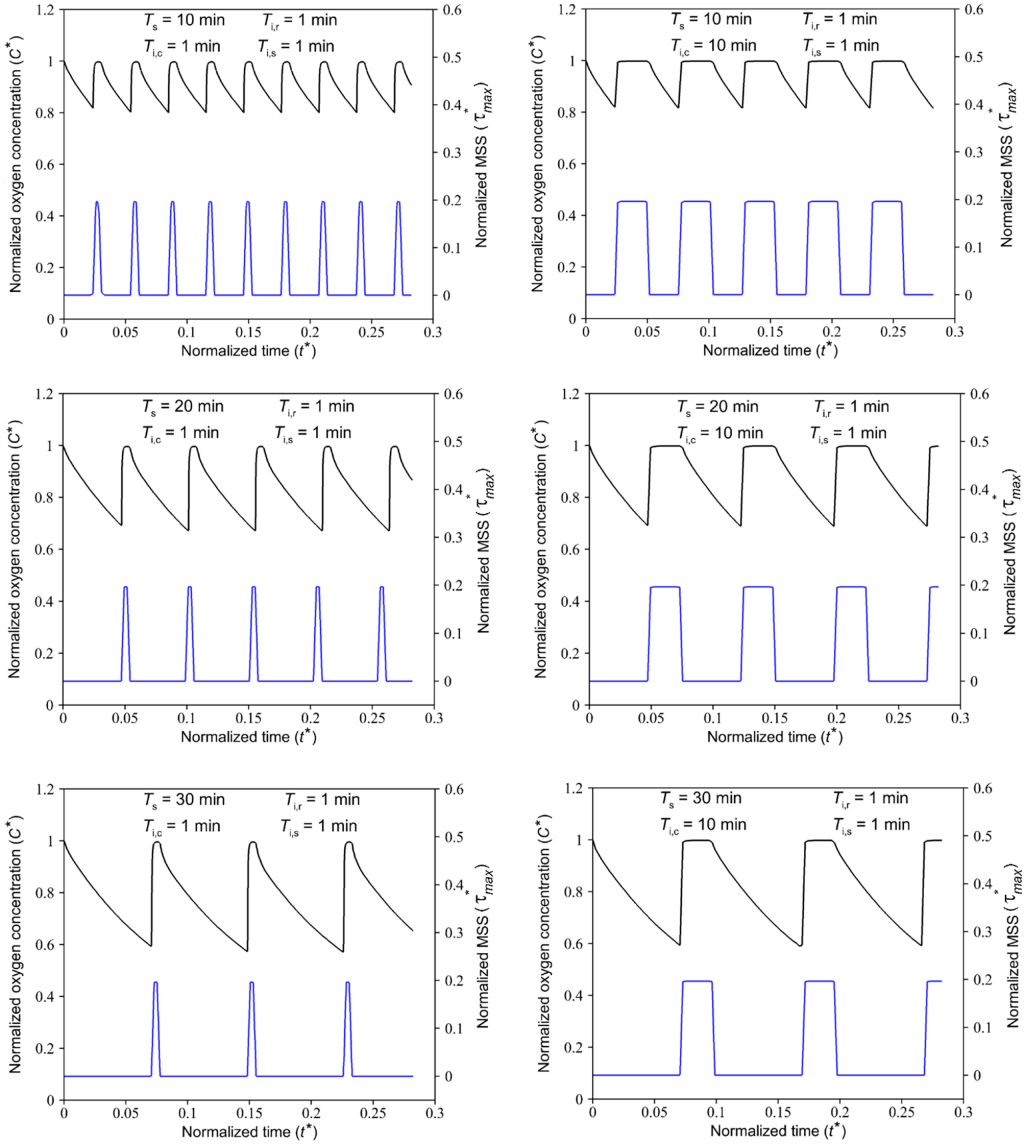
Normalized oxygen concentration and MSS in the COC microfluidic device for different medium fluid injection schemes (${C}^{*}$: Black line and ${{\tau }^{*}}_{max}$: Blue line) during HEP culture. The channel height is considered equal to $300 \mu m$

**Fig. 10:**
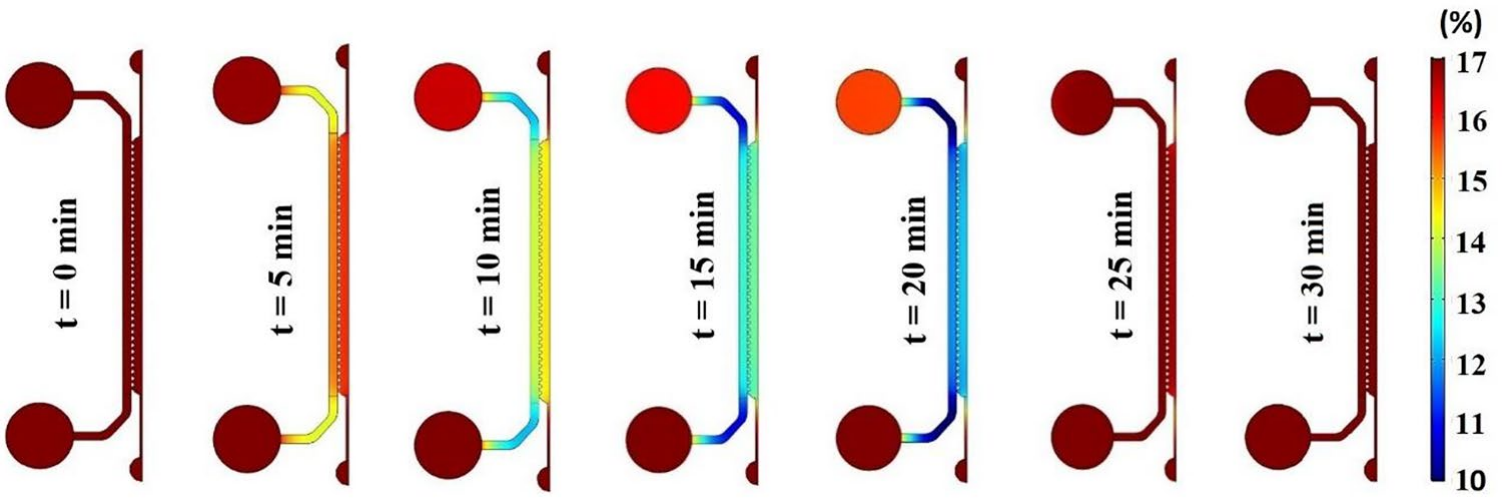
Heat map of transient oxygen concentration in the COC microfluidic device over the HEP culture in a duration of 30 min with ${T}_{\mathrm{s}}=20\mathrm{ min}$, ${T}_{\mathrm{i},\mathrm{c}}=10\mathrm{ min}$, and ${T}_{\mathrm{i},\mathrm{r}}=1\mathrm{ min}$

**Fig. 11:**
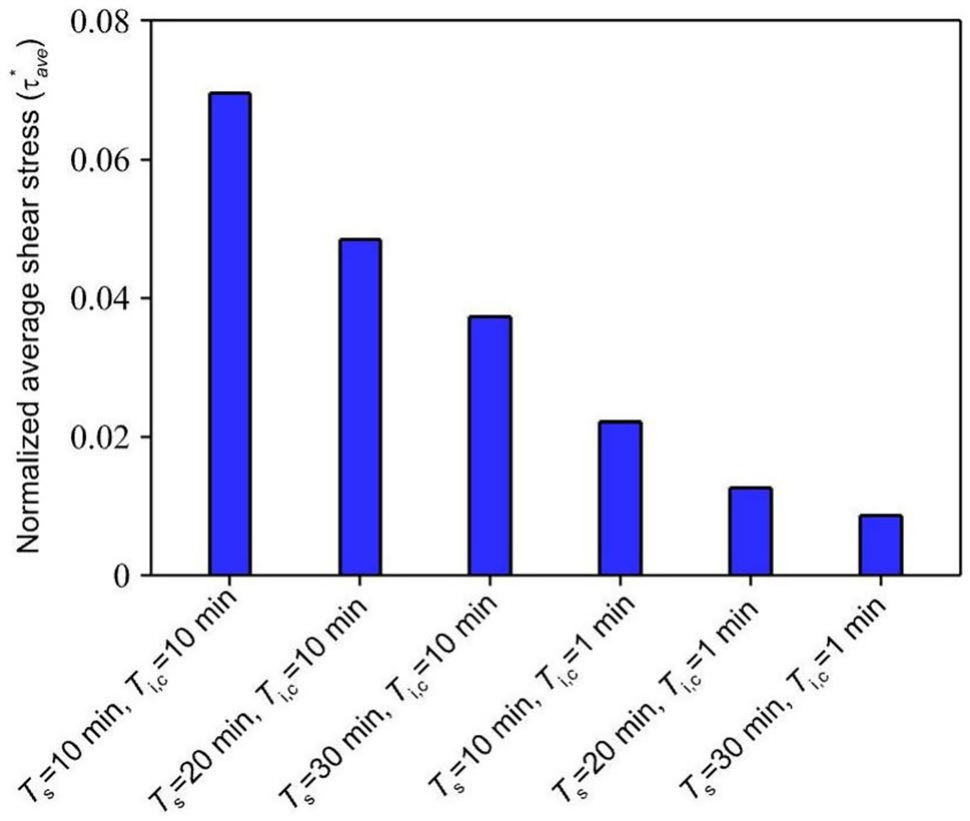
Normalized average shear stress applied on the cell layer in the COC microfluidic device for medium fluid injection with different stagnation times and continuity times (${T}_{\mathrm{i},\mathrm{r}}={T}_{\mathrm{i},\mathrm{s}}=1\mathrm{ min}$)

Along with finding a sufficient medium flow supplying the required oxygen, its damaging effect in terms of shear stress on cells has always been a concern. Although dimensional and geometrical properties of microfluidic devices could significantly affect and reduce medium-induced shear stress, some technical and biological restrictions may exist. The present study introduces a pulsatile medium flow injection pattern to minimize the destructive effect of applied fluid shear stress on the cell layer, Fig. 8. Indeed, the stop-and-flow approach injects the medium into the device only when the oxygen concentration decreases to critical values according to the desired hypoxic conditions. Therefore, the duration of the applied shear stress on the cells would decrease based on the injection stagnation time $, {T}_{\mathrm{s}}$. A suitable medium injection pattern could be considered according to “normoxic” limits and the role of shear stress on the particular cell type. To find the effect of the pulsatile medium flow on the oxygen level and the applied shear stress, non-dimensional oxygen concentration and MSS for various injection patterns were investigated, Fig. 9. Figure 10 shows a heat map of transient oxygen concentration in the medium and gel channels of the COC microfluidic device over the HEP culture to provide more insights into oxygen fluctuation in the chip. We observed that increasing the injection stagnation time decreases the minimum oxygen concentration level in the media channel, and the number of times that the cells experience high MSS reduces. Note that depletion in oxygen level during the stagnation time can affect key parameters such as cell growth, differentiation, and signaling. The oxygen concentration should be maintained within physiologically relevant limits to maintain culture viability, experimental validity, and reproducibility. Therefore, it is possible to select a pattern with a long stagnation time to culture cells that can stay alive at a low oxygen level. On the other hand, it is imperative that shear stress, as another affecting factor on the viability of the cells, be consistently kept in lower intensity. In addition to the MSS, the average of applied shear stress per cycle on the cell layer is evaluated as follows:

12 $${\tau }_{ave}=\frac{1}{t}\int \left(\frac{1}{A}\int \tau\ dA \right)dt$$

where $\tau$ and $A$ are the shear stress and the area of the cell layer, respectively. As shown in Fig. 11, the average of the applied shear stress per cycle declines with increasing the stagnation time and reducing the continuity time. The comparison of the injection models shows that the short total injection time of $3\mathrm{ min}$ is sufficient to increase the oxygen concentration up to $\sim 17\mathrm{\%}$, even for the case of maximum stagnation time. However, we should take into account that by increasing the ${T}_{\mathrm{i},\mathrm{c}}$, cells experience oxygen concentration at the maximum value for a long time. The proposed pulsatile medium flow injection can be used for mechanotransduction, i.e., deciphering the role of shear stress (as a mechanical stimulus) on cell behavior. Mainly, this platform is well-suited for cancer studies where the effect of shear stress is more critical than oxygen concentration. Oxygen concentration is less critical for cancer cells as they need much lower oxygen than normal cells and can survive under hypoxic conditions (McKeown 2014). In fact, the hypoxic condition significantly increases the epithelial-mesenchymal transition of cancer cells, causing these cells to metastasize to the other organs through lymph nodes or blood vessels (Tam et al. 2020). These circulating tumor cells are subject to the pulsatile blood flow shear stress that ultimately affect their metastatic behavior and their ultimate fate (Huang et al. 2018). Therefore, the proposed pulsatile medium flow injection can provide a systematic approach to study the effect of pulsatile shear stress on the metastatic behavior of cancer cells. Therefore, a short injection time would be a good choice when the shear stress is more critical than the oxygen concentration.

**Fig. 12:**
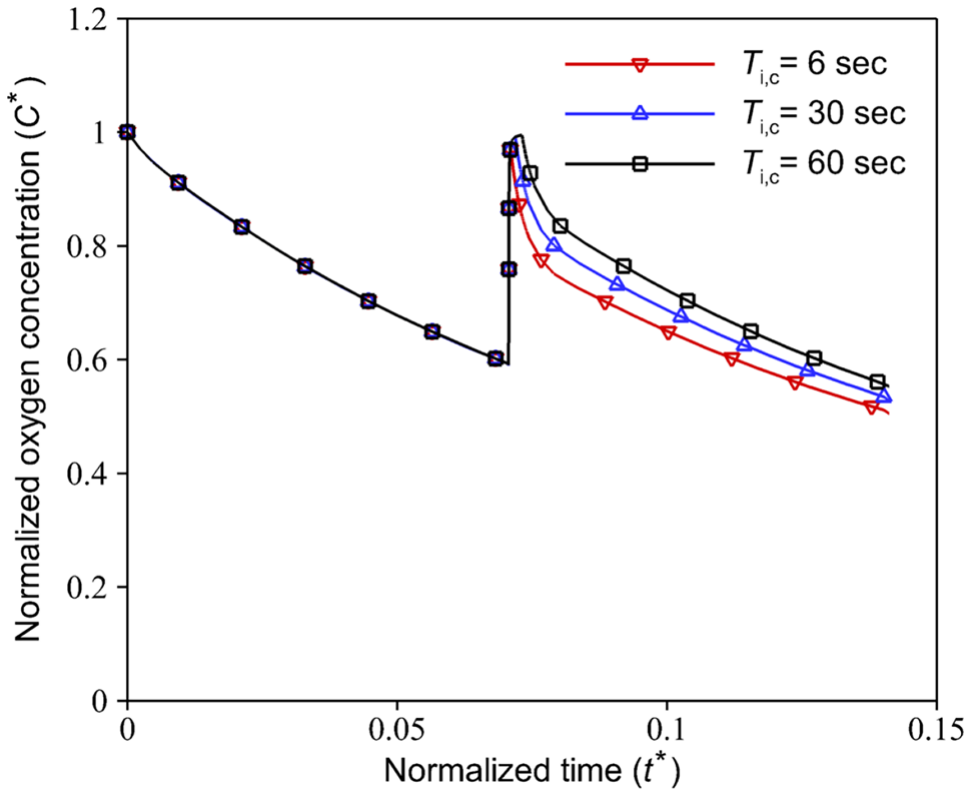
Effect of short rising time (${T}_{\mathrm{s}}=30\mathrm{ min}$ and ${T}_{\mathrm{i},\mathrm{r}}=3\mathrm{ sec}$) on the oxygen level in the COC microfluidic device during HEP culture with three different values of injection continuity time, ${T}_{\mathrm{i},\mathrm{c}}$. Results are presented over $60\mathrm{ min}$ of the medium fluid injection

**Fig. 13:**
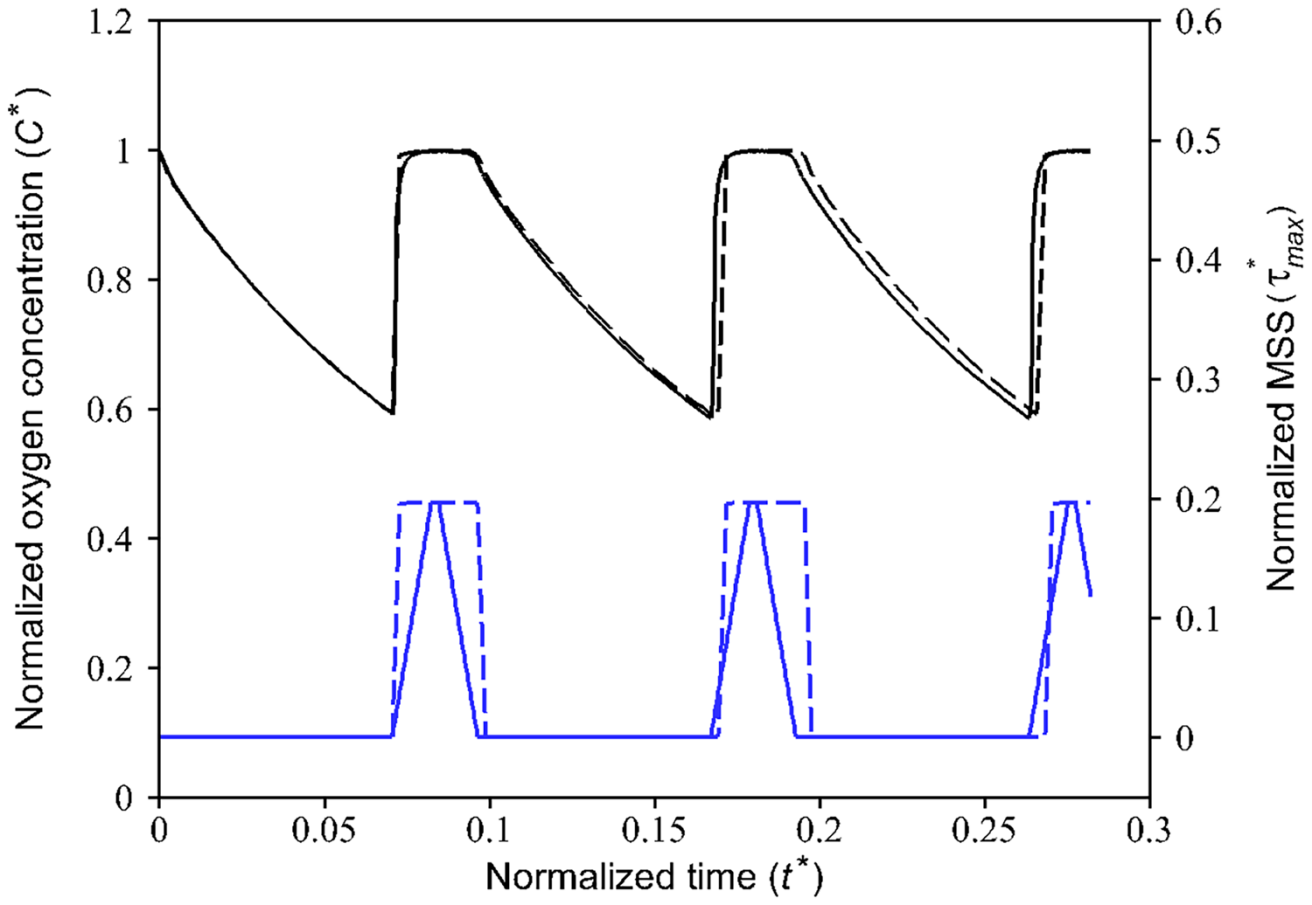
Comparison of normalized oxygen concentration and MSS in the COC microfluidic device during HEP culture for two cases with different injection rising times. For one case, it is assumed that ${T}_{\mathrm{i},\mathrm{r}}={T}_{\mathrm{i},\mathrm{s}}=5\mathrm{min}$ and ${T}_{\mathrm{i},\mathrm{c}}=1\mathrm{min}$ (${C}^{*}$: Black solid line and ${\tau }_{max}^{*}$: Blue solid line). For the other case, ${T}_{\mathrm{i},\mathrm{r}}={T}_{\mathrm{i},\mathrm{s}}=1\mathrm{min}$ and ${T}_{\mathrm{i},\mathrm{c}}=10\mathrm{min}$ are considered (${C}^{*}$: Black dash line and ${\tau }_{max}^{*}$: Blue dashed line)

In all cases, ${T}_{\mathrm{i},\mathrm{r}}$ is considered equal to $1\mathrm{ min}$ with the aim that the medium flow rate would be increased slowly in the microchannel, and cells do not experience the shear stress suddenly. However, a very short ${T}_{\mathrm{i},\mathrm{r}}$ (for instance, $3s$) could be able to increase the oxygen concentration near to $17\mathrm{\%}$, Fig. 12. To ensure the increase of oxygen concentration in the channel up to $17\%$ and prevent further oxygen loss in the subsequent cycles, selecting a sufficient ${T}_{\mathrm{i},\mathrm{c}}$, especially in the medium injection with a long stagnation time, is necessary. In the other case, injection rising time is increased instead of the injection continuity time to keep cells at a high oxygen level for a long time. We assumed that ${T}_{\mathrm{i},\mathrm{r}}={T}_{\mathrm{i},\mathrm{s}}=5\mathrm{ min}$ and ${T}_{\mathrm{i},\mathrm{c}}=1 \mathrm{min}$, which ${T}_{\mathrm{i}}$ is equal to $11\mathrm{ min}$. The obtained results for oxygen concentration and MSS are compared with the results for the case with ${T}_{\mathrm{i},\mathrm{r}}={T}_{\mathrm{i},\mathrm{s}}=1\mathrm{ min}$, and ${T}_{\mathrm{i},\mathrm{c}}=10 \mathrm{min}$ (i.e., ${T}_{\mathrm{i}}=12 \mathrm{min}$), Fig. 13. The oxygen concentration in the device for these two cases is very close. However, the average of the applied MSS on the cells in the case with a long rising time is lower. Although the medium fluid flow is injected very slowly into the channel by increasing the injection rising time, the amount of flow rate is sufficient to overcome the oxygen depletion in the device. According to Fig. 6, an extreme case of a very low flow rate can reduce the oxygen concentration.

## Conclusion

Due to the importance of oxygen availability within microfluidic cell culture devices for cell survival and proliferation, it is imperative to control the oxygen concentration in a cell culture chamber. In this work, we numerically measured the effects of various control parameters on the transient oxygen concentration in a popular commercial microfluidic device containing a thin cell layer. Consequently, the effects of changing the media flow rate and microchannel dimension were systematically characterized. Gaining an accurate knowledge of mass transport by convection and diffusion allows for effectively design a microfluidic device to provide specific oxygen concentration distributions. We found that increasing the media channel height could prevent oxygen depletion in diffusion mass transfer. Moreover, we discovered that minimum medium flow rates of $1.2 \times {10}^{-9}[{\mathrm{m}}^{3}/\mathrm{s}]$ and $2.5 \times {10}^{-10} [{\mathrm{m}}^{3}/\mathrm{s}]$ serve as a secure solution to supply the required oxygen for HEP and EC cells in the COC microdevice, respectively. Furthermore, we investigated the destructive effects of medium fluid flows in terms of applied shear stress on the cells. Although all obtained values for maximum shear stress were in the acceptable range, we tried to decrease its applied duration on the cells by introducing a pulsatile medium flow injection pattern. According to the defined and acceptable hypoxic conditions for different cell types, a specific medium injection pattern could be considered by varying stagnation and injection times of the fluid flow. Such a pulsatile flow only injects the medium to the device when the oxygen concentration decreases to critical values. This study helps to analyze the effect of different pulsatile fluid injection patterns on the oxygen level and their destructive effects on the cells, with no need for expensive and laborious laboratory experiments.
